# Associations of protein intake, sources and distribution on muscle strength in community-dwelling older adults living in Auckland, New Zealand

**DOI:** 10.1017/jns.2023.76

**Published:** 2023-08-23

**Authors:** Anne N. Hiol, Pamela R. von Hurst, Cathryn A. Conlon, Kathryn L. Beck

**Affiliations:** School of Sport, Exercise and Nutrition, Massey University, North Shore City 0632, New Zealand

**Keywords:** Muscle strength, Older adults, Protein distribution, Protein intake, Protein sources

## Abstract

Protein intake, sources and distribution impact on muscle protein synthesis and muscle mass in older adults. However, it is less clear whether dietary protein influences muscle strength. Data were obtained from the Researching Eating Activity and Cognitive Health (REACH) study, a cross-sectional study aimed at investigating dietary patterns, cognitive function and metabolic syndrome in older adults aged 65–74 years. Dietary intake was assessed using a 4-d food record and muscle strength using a handgrip strength dynamometer. After adjusting for confounders, in female older adults (*n* 212), total protein intake (*β* = 0⋅22, *P* < 0⋅01); protein from dairy and eggs (*β* = 0⋅21, *P* = 0⋅03) and plant food sources (*β* = 0⋅60, *P* < 0⋅01); and frequently consuming at least 0⋅4 g/kg BW per meal (*β* = 0⋅08, *P* < 0⋅01) were associated with higher BMI-adjusted muscle strength. However, protein from meat and fish intake and the coefficient of variance of protein intake were not related to BMI-muscle strength in female older adults. No statistically significant associations were observed in male participants (*n* = 113). There may be sex differences when investigating associations between protein intake and muscle strength in older adults. Further research is needed to investigate these sex differences.

## Introduction

Proteins are the most abundant component of skeletal muscle mass. Approximately 40 % of the body weight of a healthy adult weighing 70 kg is skeletal muscle mass, which is composed of about 20 % muscle protein^(1)^. With aging, the muscle's ability to stimulate protein synthesis is reduced, leading to a decline in muscle mass in older adults^(2–4)^. The loss of muscle mass is suggested to be a key contributor to the decrease in muscle strength observed with age, and low muscle strength is recognised as the single largest intrinsic risk factor for falls^(5,6)^.

The cause of muscle protein synthesis (MPS) impairment with aging is unknown, but it is assumed to be affected by impairments in several physiological processes^(7–12)^. It was initially thought that a reduced rate of amino acid absorption into the bloodstream and/or digestibility in the stomach and the small intestine may limit the availability of amino acids for MPS^(11,12)^. Further research suggested that a decline in amino acid transporters, which mediate the transfer of amino acids into and out of cells, may decrease amino acids that are delivered to the muscle and their subsequent uptake by the muscle^(8–10)^. Amino acid transfer and availability are both regulated by the quality and quantity of protein consumed per meal and per day.

The quality of protein in various foods is determined by their essential amino acid composition, total amount of each amino acid and relative ease of digestion in the stomach and small intestine, as well as absorption. High-quality animal proteins (e.g., meat, fish, dairy and eggs) have a greater ability to enhance MPS and increase muscle mass than plant-based proteins (e.g., soya, pea and wheat)^(13–16)^. Isanejad *et al.* reported higher total and animal (but not plant) protein intakes were associated with greater muscle strength in women aged 65–71 years^(17)^. These findings were subsequently confirmed in women and men by McLean *et al.*^(18)^. However, these studies did not consider whether the source of animal protein, i.e., meat, fish, dairy or eggs, makes any difference. In terms of protein quality properties, not all animal-based protein sources are the same. Leucine, for instance, makes up 10⋅9 % of all essential amino acids in milk proteins, compared to 8⋅8 % in beef proteins; and compared to beef/fish, egg proteins have the highest value for protein digestibility^(15,19,20)^. A highly digestible protein source offers a higher proportion of absorbed amino acids, whereas a higher leucine content indicates that less protein from a given source is necessary to maximise MPS rates^(21,22)^. As a result, the association between muscle strength and animal proteins may depend on the type of animal protein being consumed.

In older adults, a protein intake of 0⋅4 g/kg body weight (BW) per meal has been found to provide a pool of available amino acids that stimulate muscle protein maximally^(23,24)^. Therefore, it is hypothesised that eating three meals per day, with a regular protein intake of 0⋅4 g/kg BW per meal, could increase MPS throughout the day, potentially increasing and preserving muscle strength in older adults. In a randomised controlled trial, Mamerow *et al.* demonstrated that, in healthy adults aged 25–55 years, MPS was approximately 25 % higher in those with an even distribution of daily protein intake (0⋅4 g/kg BW per meal) compared with those who had a skewed meal distribution but the same amount of daily protein (1⋅2 g/kg BW per day)^(25)^. The distribution of protein is a relatively new concept. In previous studies, distribution is estimated as the coefficient of variance (CV) of the protein intake or the number of meals exceeding 0⋅4 g/kg BW. There are conflicting findings regarding the influence of protein distribution on muscle strength, which could be impeded by different distribution calculations.

The aim of this study was to examine protein intake, sources and different estimates of protein distribution throughout the day and associations with muscle strength, in community-living older adults in Auckland, New Zealand (NZ).

## Materials and methods

### Study design

Data for this study were obtained from Researching Eating, Activity and Cognitive Health (REACH) study; the methodology has been described elsewhere^(26)^. In brief, REACH participants were adults aged between 65 and 74 years, proficient in English, and living independently (i.e., not in residential care) in Auckland, NZ. Ineligibility criteria included a diagnosis of dementia or any condition which may impair cognitive function (e.g., previous head injury, stroke), taking medication which may influence cognitive function, colour blindness (due to cognitive testing requirements) or experiencing any other event in the last 2 years which may have had a substantial impact on dietary intake or cognition.

The research protocol was approved by the Massey University Human Ethics Committee: Southern A, Application 17/69 and all participants provided written informed consent.

### Data collection

Age, sex, polypharmacy and smoking data were collected using a health and demographic written questionnaire. Physical activity was assessed through the International Physical Activity Questionnaire – short form^(27)^. A physical activity score was calculated using the metabolic equivalent of a task (MET-min) where 1 min of walking, moderate or vigorous activity equates to 3⋅3, 4⋅0 or 8⋅0 METs, respectively^(27)^. Polypharmacy was defined as five or more daily medications, and smoking was described as both current and previous smoking^(28)^.

Weight (in cm, to the nearest 0⋅1 cm) was measured using a stadiometer (SECA). BW (in kg, to the nearest 0⋅1 kg) was assessed using floor scales (Wedderburn). Body mass index (BMI) was calculated as weight in kilograms divided by height in metres squared.

Muscle strength was assessed using an adjustable handgrip strength dynamometer (JAMAR HAND). The handgrip dynamometer measures the maximum kilograms of force per trial, where three trials were undertaken for each of the right and left hands with a 15–20 s break between trials^(29,30)^. The mean of three trials for each hand was noted and the highest value of the two means was considered the final value. Because the Foundation for the National Institutes of Health (FNIH) showed that BMI-standardised handgrip strength is more strongly associated with falls and related injuries than absolute handgrip strength, we used handgrip strength adjusted for BMI^(31)^. Low muscle strength was defined as BMI-handgrip strength <1⋅00 m^2^ in men and <0⋅56 m^2^ in women^(32)^.

Dietary intake data were collected using a 4-d food record. Participants were asked to write down everything they ate and drank for four consecutive days, including at least one weekend day. They were advised to describe the amount of food consumed using household measurements. The dietary data collection method has been described in detail elsewhere^(33)^.

### Data handling

Food record data were entered by trained nutritionists into *FoodWorks 10*, which is based on the New Zealand Food Composition database^(34,35)^. Energy and macronutrient intake was generated as kilojoules and grams of intake per day, respectively. Relative protein intake was calculated by dividing the amount of protein (g) consumed by BW (kg).

Meals were categorised into ‘breakfast’, the ‘mid-day meal’ and the ‘evening meal’ based on the time of day at which protein was consumed. Protein in g and g/kg BW was calculated at breakfast, the mid-day meal and the evening meal. The consumption of 0⋅4 g/kg BW of protein at each meal was reported. Protein distribution was calculated for each participant as a:

– CV ( = standard deviation of protein intake of the three meals (g)/total protein intake for the three meals (g)). A CV of zero indicates that protein is distributed evenly throughout the day. The smaller the CV, the more even the distribution and,
– The frequency of consuming ≥0⋅4 g/kg BW of protein across the day's meal. This was calculated by adding the number of meals at which individuals consumed ≥0⋅4 g/kg BW of protein. The value for the frequency of protein consumption variable ranged from 1 to 3 (breakfast, the mid-day and the evening meal). A higher number represented a more even distribution.

All foods from the food records were allocated into one of 27 food groups based on the main sources of protein intake reported in the 2008/09 New Zealand Adult Nutrition Survey^(36)^. The food groups were further classified under meat and fish; dairy and egg products; and plants according to the main type of protein they contained. Protein intake from meat and fish; dairy and egg products; and plants were calculated in g/kg BW.

### Statistical analysis

All continuous variables were tested for normality with the Shapiro–Wilk test. Participant characteristics were displayed as median and interquartile range (IQR) for non-parametric continuous variables and as counts with percentages for categorical variables. Differences between groups were tested using the Mann–Whitney *U* test for non-normally distributed continuous data or the *χ*^2^ test for categorical variables. Multiple regression analysis was performed separately in females and males to examine associations between BMI-muscle strength (dependent variable) and:

– Relative protein intake g/kg BW per day. The confounding variables were energy intake (kJ), physical activity level (MET-min/week), age (years), polypharmacy status (yes or no) and smoking status (yes or no).
– Protein from meat and fish (g/kg BW); dairy and egg products (g/kg BW); and plants (g/kg BW). These models accounted for the effects of energy intake (kJ), physical activity level (MET-min/week), age (years), polypharmacy status (yes or no) and smoking status (yes or no). For these models, protein intakes from meat and fish; dairy and egg products; and plants were included in the same regression model to adjust for one another.
– Different estimates of protein distribution throughout the day (CV and frequency consuming of meals containing ≥0⋅4 g/kg BW of protein per day). The models were controlled for total protein intake (g), energy intake (kJ), physical activity level (MET-min/week), age (years), polypharmacy status (yes or no) and smoking status (yes or no).

All statistical analyses were performed using IBM SPSS Statistics for Windows, version 27.0 (IBM SPSS, Armonk, NY, USA). All the probability values were two-tailed and were considered significant if a *P*-value was <0⋅05.

## Results

A total of 371 individuals participated in the REACH study. We excluded individuals who did not provide or had incomplete variables such as food records (*n* 44), handgrip strength (*n* 1) and physical activity (*n* 1). The final data set included 325 older adults (113 males and 212 females). Table 1 presents descriptive statistics according to sex for the study population. There were no significant differences between males and females in physical activity (*P* = 0⋅88), polypharmacy (*P* = 0⋅31) or smoking status (*P* = 0⋅93). Females were younger (*P* < 0⋅01) and had a lower height (*P* < 0⋅01), weight (*P* < 0⋅01), BMI (*P* = 0⋅02) and BMI-muscle strength (*P* < 0⋅01) compared with males. Males had a higher prevalence of low BMI-muscle strength (4⋅4 %) than females (3⋅8 %).

**Table 1.** Participants’ characteristics

Characteristics	Total		Females		Males		*P*-value
*n* (%)^a^	325		212	(65⋅2)	113	(34⋅8)	<0⋅01**
Age, years^b^	69⋅7	(67⋅9, 71⋅6)	69⋅3	(67⋅5, 71⋅4)	70⋅3	(68⋅5, 71⋅9)	0⋅01*
Physical activity, MET min/week^b^	3088⋅0	(1679⋅5, 5079⋅0)	3126⋅0	(1618⋅0, 5121⋅0)	3066⋅0	(1778⋅7, 4942⋅0)	0⋅88
Height, m^b^	1⋅7	(1⋅6, 1⋅7)	1⋅6	(1⋅6, 1⋅7)	1⋅8	(1⋅7, 1⋅8)	<0⋅01**
Body weight, kg^b^	71⋅2	(63⋅2, 81⋅5)	67⋅0	(59⋅6, 74⋅7)	81⋅5	(73⋅7, 89⋅3)	<0⋅01**
BMI, kg/m^2b^	25⋅6	(23⋅1, 28⋅2)	25⋅3	(22⋅7, 28⋅1)	26⋅4	(24⋅3, 28⋅3)	0⋅02*
Polypharmacy status, Yes, *n* (%)^a^	25	(7⋅7)	14	(6⋅6)	11	(9⋅7)	0⋅31
Smoker status, Yes *n* (%)^a^	71	(21⋅8)	46	(21⋅7)	25	(22⋅1)	0⋅93
BMI-muscle strength^b^	1⋅1	(0⋅9, 1⋅4)	1⋅0	(0⋅8, 1⋅1)	1⋅6	(1⋅3, 1⋅8)	<0⋅01**
Prevalence of low BMI-muscle strength^a^	13	(4)	8	(3⋅8)	5	(4⋅4)	<0⋅01**

^a^Categorical values are expressed as frequency (percentage). Differences between groups were tested using the Mann–Whitney *U* test for non-normally distributed continuous data or the *χ*^2^ test for categorical variables. Sex difference at **P < 0⋅01, *P < 0⋅05.

^b^Continuous values are expressed as median (25th, 75th percentile).

Table 2 shows the median protein intake, distribution and sources for both females and males. Females consumed significantly less energy (*P* < 0⋅01), carbohydrate (*P* < 0⋅01), fat (*P* < 0⋅01) and absolute protein (*P* < 0⋅01) than males. Females had a similar relative protein intake per day (1⋅1 (0⋅9, 1⋅3) g/kg BW) to males (1⋅2 (0⋅9, 1⋅4) g/kg BW) (*P* = 0⋅38).

**Table 2.** Median protein intake, distribution and sources

		Total		Females		Males		*P*-value
Energy intake, kJ/d^a^		7914⋅7	(6717⋅1, 9271⋅3)	7434⋅3	(6339⋅3, 8382⋅0)	9239⋅4	(7920⋅1, 10 729.9)	<0⋅01**
Carbohydrate, g/d^a^		86⋅4	(147⋅1, 225⋅9)	172⋅9	(136⋅2, 201⋅2)	222⋅9	(185⋅2, 262⋅6)	<0⋅01**
Total fat, g/d^a^		76⋅7	(63⋅6, 94⋅1)	72⋅9	(61⋅5, 86⋅5)	90⋅8	(69⋅5, 107⋅7)	<0⋅01**
Protein, g/d^a^		79⋅4	(67⋅5, 97⋅2)	75⋅0	(63⋅5, 88⋅2)	95⋅9	(77⋅5, 107⋅2)	<0⋅01**
Protein, g/kg BW per day^a^		1⋅1	(0⋅9, 1⋅4)	1⋅1	(0⋅9, 1⋅3)	1⋅2	(0⋅9, 1⋅4)	0⋅38
Protein intake at each meal, g/kg BW^a^	Breakfast	0⋅3	(0⋅2, 0⋅3)	0⋅2	(0⋅2, 0⋅3)	0⋅3	(0⋅2, 0⋅4)	0⋅01*
	Mid-day	0⋅3	(0⋅2, 0⋅4)	0⋅3	(0⋅3, 0⋅4)	0⋅3	(0⋅2, 0⋅4)	0⋅14
	Evening	0⋅5	(0⋅3, 0⋅7)	0⋅5	(0⋅4, 0⋅7)	0⋅6	(0⋅4, 0⋅7)	0⋅30
Number of meals providing ≥ 0⋅4 g/kg BW of protein^b^	0	44	(13⋅5 %)	25	(11⋅8 %)	19	(16⋅8 %)	0⋅53
	1	188	(57⋅8 %)	126	(59⋅4 %)	62	(54⋅9 %)	
	2	77	(23⋅7 %)	51	(24⋅1 %)	26	(23⋅0 %)	
	3	16	(4⋅9 %)	10	(4⋅7 %)	6	(5⋅3 %)	
CV^a^		0⋅5	(0⋅3, 0⋅6)	0⋅5	(0⋅3, 0⋅6)	0⋅5	(0⋅3, 0⋅7)	0⋅96
Protein source, g/kg BW^a^	Protein from meat and fish	0⋅4	(0⋅3, 0⋅5)	0⋅4	(0⋅3, 0⋅5)	0⋅4	(0⋅3, 0⋅6)	0⋅09
	Protein from dairy and eggs	0⋅3	(0⋅2, 0⋅4)	0⋅3	(0⋅2, 0⋅4)	0⋅2	(0⋅2, 0⋅3)	0⋅19
	Plant protein	0⋅4	(0⋅3, 0⋅6)	0⋅4	(0⋅3, 0⋅6)	0⋅5	(0⋅4, 0⋅6)	0⋅28

^a^Continuous values are expressed as median (25th, 75th percentile).

^b^Categorical values are expressed as frequency (percentage). Sex difference at **P < 0⋅01, *P < 0⋅05.

The evening meal provided the highest median protein intake (g/kg BW) at 0⋅5 (0⋅3, 0⋅7), followed by the mid-day meal at 0⋅3 (0⋅2, 0⋅4), and breakfast at 0⋅3 (0⋅2, 0⋅3). Protein distribution was uneven throughout the day as indicated by a median CV for protein distribution of 0⋅5 (0⋅3, 0⋅6) for both males and females. When comparing individual meals to the 0⋅4 g/kg threshold, 5 % of females and 5 % of males met the threshold for all three meals. Males and females had a median protein intake (g/kg BW) of 0⋅4 (0⋅3, 0⋅6) from plant sources, 0⋅4 (0⋅3, 0⋅5) from meat and fish sources, and 0⋅3 (0⋅2, 0⋅4) from dairy and egg sources.

Table 3 shows the association of relative protein intake, sources and distribution on BMI-muscle strength in females. Relative protein intake was positively associated with BMI-muscle strength (*β* = 0⋅22, *P* < 0⋅01). Protein from dairy and egg products (*β* = 0⋅21, *P* = 0⋅03), as well as plant proteins (*β* = 0⋅60, *P* < 0⋅01), but not with proteins from meat and fish (*β* = 0⋅04, *P* = 0⋅55) was positively associated with BMI-muscle strength. The CV was not significantly associated with BMI-muscle strength (*β* = −0⋅05, *P* = 0⋅47), whereas meal frequency of 0⋅4 g/kg BW was significantly associated with BMI-muscle strength (*β* = 0⋅08, *P* < 0⋅01).

**Table 3.** Association between protein intake and muscle strength in females

BMI-muscle strength (dependent variable)	Coefficient (*β*)	Standard error (*B*)	*P*-value
Model total protein intake			<0⋅01**
Constant	0⋅98	0⋅41	0⋅02*
Protein intake, g/kg BW per day	0⋅22	0⋅05	<0⋅01**
Model sources of protein			<0⋅01
Constant	1⋅10	0⋅39	0⋅01
Protein from meat and fish, g/kg BW per day	0⋅04	0⋅06	0⋅55
Protein from dairy and eggs, g/kg BW per day	0⋅21	0⋅10	0⋅03
Protein from plant, g/kg BW per day	0⋅60	0⋅09	<0⋅01
Model 1 protein distribution			<0⋅01
Constant	1⋅07	0⋅42	0⋅01
Number of meals providing at least 0⋅4 g/kg BW per day	0⋅08	0⋅02	<0⋅01
Model 2 protein distribution			<0⋅01
Constant	1⋅01	0⋅43	0⋅02
CV of protein intake at main meals	−0⋅05	0⋅07	0⋅47

CV, coefficient of variance of protein intake (g) at breakfast, mid-day meal and evening meal.

Significant at **P < 0⋅01, *P < 0⋅05.

Table 4 shows there was no relationship between total protein intake (*β* = 0⋅11, *P* = 0⋅23), proteins from meat and fish (*β* = 0⋅06, *P* = 0⋅65), from dairy and egg products (*β* = 0⋅00, *P* = 0⋅99) or plant proteins (*β* = 0⋅32, *P* = 0⋅10) and BMI-muscle strength in males. No association was observed CV of protein across main meals (*β* = −0⋅21, *P* = 0⋅12) and number of meals containing at least 0⋅4 g of protein/kg BW (*β* = −0⋅05, *P* = 0⋅35) and BMI-muscle strength in males.

**Table 4.** Association between protein intake and muscle strength in males

BMI-muscle strength (dependent variable)	Coefficient (*β*)	Standard error (*B*)	*P*-value
Model total protein intake			<0⋅01**
Constant	1⋅75	0⋅89	0⋅05
Protein intake, g/kg BW	0⋅11	0⋅11	0⋅23
Model sources of protein			<0⋅01
Constant	1⋅71	0⋅90	0⋅06
Protein from meat and fish, g/kg BW per day	0⋅06	0⋅14	0⋅65
Protein from dairy and eggs, g/kg BW per day	0⋅00	0⋅24	0⋅99
Protein from plant, g/kg BW per day	0⋅32	0⋅20	0⋅10
Model 1 protein distribution			<0⋅01
Constant	1⋅83	0⋅94	0⋅05
Number of meals providing at least 0⋅4 g/kg BW per day	−0⋅05	0⋅05	0⋅35
Model protein distribution			<0⋅01
Constant	2⋅01	0⋅90	0⋅03
CV of protein intake at main meals	−0⋅21	0⋅13	0⋅12

CV, coefficient of variance of protein intake (g) at breakfast, mid-day meal and evening meal. Significant at **P < 0⋅01, *P < 0⋅05.

## Discussion

In this cross-sectional study, we investigated the relationship between protein intake, sources and distribution and BMI-muscle strength in females and males older adults living in Auckland, NZ. In females, the findings indicate that BMI-muscle strength was associated with relative protein intake. This relationship was independent of total energy intake, age, physical activity, smoking and polypharmacy status. We also demonstrated an association of protein intake from dairy, eggs and plant sources; but not with protein from meat and fish intake and BMI-muscle strength. We found the CV of protein intake was not related to BMI-muscle strength. However, we demonstrated that a greater frequency of protein consumption of 0⋅4 g/kg BW per meal was associated with BMI-muscle strength. In male older adults, there were no associations between protein intake, sources or distribution and BMI-muscle strength.

### Associations of protein intake, sources and distribution on BMI-muscle strength in females

We provide evidence that relative protein intake is positively associated with BMI-muscle strength in females older adults aged between 65 and 74 years. Our findings are consistent with previous cross-sectional studies indicating an association between relative protein intake and muscle strength adjusted by BW or BMI in older adults^(37–39)^. In contrast, other studies found that there was no association between relative protein intake and muscle strength^(40–42)^. One explanation for their lack of associations could be related to not adjusting muscle strength for BW or BMI.

Muscle strength-adjusted BMI or BMI-muscle strength has been proposed as the ideal marker for muscle strength because it minimises the confounding effect of BW^(43–46)^. Furthermore, low BMI-muscle strength represents not only low muscle strength but also obesity, both of which are closely related to impairment of MPS in response to ingested protein^(43–49)^. In this regard, we used BMI-muscle strength and demonstrated a relationship between relative dietary protein and muscle strength in females older adults.

In multiple regression analysis, examining associations between protein sources and BMI-muscle strength, dairy and egg proteins (but not meat and fish) were found to be positively associated with BMI-muscle strength. Because the amount of protein consumed from both animal food group sources was comparable, these findings suggest that the protein quality properties of dairy and eggs, which have a higher leucine content and higher value for protein digestibility than meat and fish proteins, may explain the association found in this study. In contrast to previous research^(17,18)^, an association of protein from plant sources on BMI-muscle strength was observed. The differences in findings could be attributed to differences in the amount of plant protein consumed. Participants in this cohort consumed a similar amount of protein from both animal and plant-based sources, whereas in other studies, the range of plant-based protein sources was much smaller.

When calculating the distribution of protein using the CV calculation, we found no association between the CV and BMI-muscle strength in females and male older adults. This result aligns with the literature^(40,50–52)^ and suggests that the CV might give information about the distribution of protein, but it does not provide information about the amount of protein consumed. Murphy *et al.* also demonstrated that MPS responses were not different in older adults subjected to uneven or even distribution^(53)^. Given this, the consumption of meals containing less than 0⋅4 g/kg BW, required for maximal MPS in older adults may help to explain this finding. We demonstrated a positive association between the frequency of meals of ≥0⋅4 g/kg BW and BMI-muscle strength in female older adults. Two recent studies also investigated associations between muscle strength and consuming at least 0⋅4 g/kg BW in older adults^(51,54)^. While both studies did not adjust muscle strength for BMI, Johnson *et al.* did account for BMI in their multiple regression analyses and found an association between absolute muscle strength and consuming at least 0⋅4 g/kg BW in females older adults^(54)^. Gingrich *et al.*, on the other hand, found no relationship between the number of meals providing 0⋅4 g/kg BW and muscle strength^(51)^. However, BMI and sex differences were not considered in their analysis. These findings confirm that the conflicting findings on the influence of protein distribution on muscle strength are impeded by different protein distribution calculations. In the wider literature, it is necessary to reach an agreement on an appropriate protein distribution calculation.

### Associations of protein intake, sources and evenness distribution on BMI-muscle strength in males

There was no association of protein intake, sources or distribution on BMI-muscle strength in older male adults. Males have a greater anabolic response to protein intake with greater muscle strength than females. Consequently, males may require more protein in order to develop greater muscle strength than do females. In this population male protein intake was comparable to females, which may explain the association between muscle strength and relative protein intake in females but not males.

One of the main strengths of this study was the use of BMI-muscle strength, which has been shown to be more strongly associated with composite adverse outcomes such as falls compared with absolute muscle strength^(31)^. An additional strength was the use of food records, which is considered the gold standard of dietary assessment methods due to their accuracy in estimating actual dietary intakes^(55)^. Relative protein intake was used to account for differences in BW, and particularly weight differences between sexes in older adults^(56)^. In addition, several variables related to protein intake (quantity, sources and distribution) were addressed concurrently in this study. Finally, adjustments were made for age, total energy intake, physical activity, polypharmacy and smoking status, all of which are important potential confounders in the relationship between muscle strength and dietary intake. Total protein intake was adjusted for when considering the distribution of protein consumed throughout the day. The study's limitations include its cross-sectional design, which limited its ability to detect causality; thus, only associations can be discussed.

## Conclusions

In this study, we demonstrated that females older adults who consumed a high relative protein intake and frequently consumed higher amounts of protein at each meal had increased BMI-muscle strength. Protein from dairy and eggs, as well as plant sources, was associated with BMI-muscle strength, but not protein from meat and fish. In male older adults, however, there was no association of protein intake, sources or distribution between BMI-muscle strength. These results indicate that there may be sex differences when investigating associations between dietary protein quantity, sources and distribution and muscle strength. Future research should investigate the factors that contribute to the sex differences in this relationship.
